# Estimating prevalence of overweight and obesity at the neighborhood level: the value of maternal height and weight data available on birth certificate records

**DOI:** 10.1186/1478-7954-8-16

**Published:** 2010-05-25

**Authors:** David A Webb, Jessica M Robbins, Joan R Bloch, Jennifer F Culhane

**Affiliations:** 1 Philadelphia Department of Public Health. Philadelphia, Pennsylvania, USA; 2 College of Nursing and Health Professions and School of Public Health at Drexel University, Philadelphia, Pennsylvania, USA; 3 Current Address: Division of Adolescent Medicine at Children's Hospital of Philadelphia, Philadelphia, Pennsylvania, USA

## Abstract

**Objective:**

To determine the value of maternal height and weight data on birth certificate records when estimating prevalence of overweight and obese adults at the neighborhood level.

**Research Design and Methods:**

Regression analysis was used to determine how much variation in the percentage of the adult population with a body mass index (BMI) of ≥ 25 (based on survey data) could be accounted for by the percentage of mothers with BMI ≥ 25 (based on birth certificate data) -- alone and in combination with other sociodemographic characteristics of census tracts.

**Results:**

Alone, the percentage of mothers with BMI ≥ 25 explained more than half (R^2 ^= .52) of the variation in the percentage of all residents in census tracts with BMI ≥ 25; in combination with several measures of the sociodemographic characteristics of the census tracts, 75% ( R^2 ^= 75.2) of the variation is explained.

**Conclusions:**

Maternal height and weight data available from birth certificate records may be useful for identifying neighborhoods with relatively high or low prevalence of adult residents who are overweight or obese. This is especially true if used in combination with readily available census data.

## Introduction

Adult obesity is known to be associated with substantial increased risks of morbidity -- including diabetes, cardiovascular disease, and arthritis, as well as several specific forms of cancer [1]. The increased health care costs, activity limitations, and reduced productivity associated with obesity have also been documented [2]. Obesity and overweight are extremely common, affecting a majority of the adult population in the United States [3]. Moreover, onset of the disease appears to be occurring at younger ages [4]. Because it is so closely tied to behavioral/modifiable risk factors, obesity is widely regarded as a potentially preventable disease [5]. Not surprisingly, therefore, it has risen into the top rank of public health concerns [6].

Adults with a body mass index (BMI; body weight in kilograms divided by square of height in meters) between 25 and 29 are typically considered to be pre-obese or overweight, while individuals with a BMI of 30 or greater are defined as obese. The risks associated with high BMI (that is, 25 or over) are generally regarded as being on a continuum as BMI increases. For that reason, the prevalence of both high BMI and obesity are routinely monitored as part of the Centers for Disease Control Behavioral Risk Factor Surveillance System.

While national surveys provide important information about the prevalence and trends of adult overweight/obesity in the US at the national and state levels, the need to strengthen local public health data systems for surveillance at the neighborhood or small-area levels has been widely recognized [7]. In brief, local public health authorities and advocates are often in need of information about their own communities in order to appropriately prioritize, develop, target, and evaluate related interventions.

In April 2000, the National Center for Health Statistics (NCHS) formally adopted a new US Standard Certificate of Live Birth (effective beginning with 2003 live births), which included maternal height and weight as reportable data elements. Revisions of the US Standard Certificate of Live Births have occurred approximately every 10 years following a thorough review process that has been described in detail elsewhere [8]. Assessing the potential usefulness of newly added birth certificate items for clinical and/or public health research is important because the hospital, state, and local resources devoted to implementing the changes are significant [8,9]. In light of this fact and given the public health significance of obesity in the US, we sought to explore the value of BMI information now being recorded on birth certificate records. Specifically, the objective was to assess whether aggregating BMI data available from birth certificate records could provide a proxy indicator of overweight/obesity prevalence for adult populations at the neighborhood or small-area levels.

## Data and methods

### Data

Maternal height and weight data available from Pennsylvania birth certificate records for all 2003-2004 Philadelphia resident live births were used to calculate the percentage of childbearing women with BMI 25 or greater (at first prenatal visit) for each of the census tracts in the city. Self-reported height and weight data from three biannual health surveys of city residents were combined and used to calculate the percentage of all adults in each census tract with BMI 25 or greater. These data were available from the 2000, 2002, and 2004 Southeastern Pennsylvania Household Health Survey, administered by the Philadelphia Health Management Corporation (PHMC). Details about the survey content, the sample design, and methodology are available elsewhere, in publications[10] and on PHMC's Web site [11]. In brief, the surveys are conducted biannually, are based on multistage probability sample, and are designed to collect a wide range of health-related information pertaining to residents of five contiguous counties, including Philadelphia. A combined total of 12,680 Philadelphia adult residents responded to the three surveys.

Both the original birth certificate and survey databases used in the analyses contained de-identified, individual-level data, but included census tract of residence as a variable. Thus, calculating tract-level variables was a relatively straightforward process of aggregating, then merging the available data from the two different database sources.

### Methods

To avoid the statistical instability inherent in estimation involving small numbers, only census tracts with 50 or more live births and 50 or more survey respondents were included in the results reported here. A total of 68 of the city's 365 census tracts met those criteria; these tracts represented, respectively, 35.2% of the 2003 resident live births and 34.7% of the survey respondent records.

Simple scatter plots and bivariate regression analysis were used to explore and quantify the relationship between the percentage of childbearing women with BMIs of 25 or greater and the percentage of all adults with BMIs of 25 or greater calculated from the survey data.

Multiple regression analysis was also used to assess the extent to which overweight/obesity derived from the height and weight data on the birth certificate records could be used in combination with tract-level population data, readily available from the U.S. Census Bureau, to approximate neighborhood-level prevalence of overweight and obesity. This seemed like a logical extension of the bivariate analysis described above, given the inherent value of ranking or estimating the prevalence of overweight and obese adults at the neighborhood level, and the fact that census tract indicators are generally accessible to public health researchers.

In preliminary analyses, stepwise multiple regression was used to identify which set of sociodemographic variables in conjunction with our area-level BMI measures best helped to explain neighborhood differences in prevalence levels. Specifically, the following variables were included in the stepwise regression analysis:

• Percent of the population non-Hispanic White

• Percent non-Hispanic African American

• Percent Hispanic

• Percent Asian

• Median age

• Median income

• Percent of population over 55 years of age

• Percentage of the population living at or below the poverty line

• Percent (25 years or older)with less than a high school degree

The results indicated that the percentage of the population over 25 years with less than a high school education, the percentage Asian, and the percentage of the population 50 years of age and older were the statistically significant and most salient census tract variables associated with our measure of the percentage of all adults with BMIs of 25 or greater, as calculated from the available survey data. These preliminary findings were consistent with the literature, which has shown that the risk of obesity increases with age, is lower among Asians than among whites, and is inversely related to socioeconomic status [12]. Only results based on models that included these parameters as independent variables, in conjunction with the percentage of childbearing women with BMIs 25 or greater, are reported here.

All analyses were conducted using SPSS 15.1 for Windows.

## Results

As Table 1 shows, the study census tracts were representative of the city's population, since the sociodemographic characteristics of residents in those tracts matched those for the city as a whole. Specifically, data from the US Census Bureau show that summary measures of income, poverty, age, education, and ethnic/racial composition for the 68 census tracts used in the analysis were almost identical to those for Philadelphia at large.

**Table 1:** Sociodemographic Characteristics of Residents in Study Census Tracts and Philadelphia Residents as a Whole.

	*Study Census Tracts*	*Philadelphia (All Tracts)*
**Median Income/yr**	$ 30,746	30,604

		

**Poverty: % of Families**	22.5	23.2

		

**Median Age: years**	34.2	34.6

		

**Education:**		

**% < High School**	28.7	28.5

		

**Ethnicity/race**		

% White*	47.4	47.5

% Black*	43.1	43.2

% Asian	4.2	4.5

% Hispanic (any race)	11.2	11.5

* Includes Hispanics who identify themselves as "White" or "Black"

**Figure 1:**
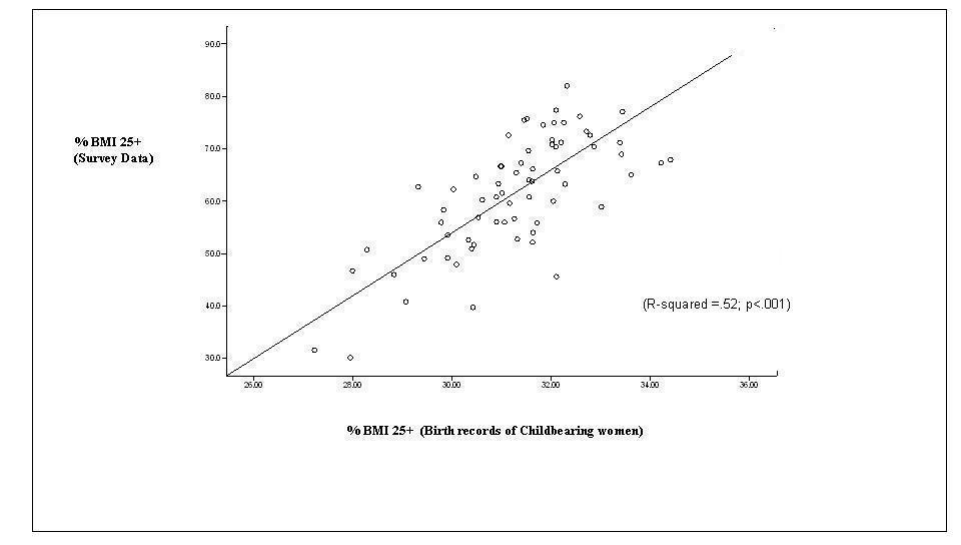
**Percentage of mothers with BMI ≥ 25 vs. percentage of all adults with BMI ≥ 25 by census tract**.

The percentage of Philadelphia resident mothers with BMI 25 or greater is plotted against the percentage of all adults with BMI 25 or greater in Figure 1. A positive linear relationship is visually evident. This is confirmed by the results of the bivariate least-squares regression analysis, which quantifies and establishes the statistical significance of this relationship. Specifically, slightly more than half of the variance in the "dependent" variable is explained by the "independent" variable in the analysis (R-squared = .52; p < .001). Thus, the findings suggest that the prevalence of childbearing women with BMI > = 25, calculated directly from the available birth certificate data, provides a reasonably good proxy for the prevalence of BMI > = 25 for all adults at the neighborhood or small-area level -- defined here in terms of standard US census tract boundaries.

The results of the multiple regression analyses, which included socioeconomic characteristics as additional independent variables, are presented in Table 2. As shown in Table 2 (model 2), the percentage of the population with less than a high school degree (among those 25 years and older), the percentage Asian, and the percentage 50 years or older together explain a total of about 59% of the variance in the percentage of the population in the census tracts with a BMI > = 25. When these variables are used in combination with our maternal BMI measure (model 3), almost 75% of the variance is explained. Thus, knowledge of the maternal BMI > = 25 explains an additional 15% of the variance in the percentage of the population in the census tracts with a BMI > = 25, over and above what is explained by the selected measures of their socioeconomic characteristics. Note that beta coefficients, which express effects in standard deviation units, can be used to compare the relative influence of variables in model 3. The beta value (.401) for the BMI measure from birth certificate records indicates that, in terms of estimating overweight and obesity prevalence at the census-tract level, the BMI measure is more salient than basic measures of socioeconomic status (percent less than high school degree) and age (percent 50 year or older). It is also at least as salient for the purposes of estimating overweight and obesity prevalence as percent Asian (Beta = .403), which proved in our preliminary analysis of the data to be the only measure of race/ethnic composition to be important to include in our models.

**Table 2:** Multiple Regression Results Estimating Percent of Census Tract Population with BMI ≥ 25.

	MODEL 1	Model 2	MODEL 3
**Independent**	Coeff(95% C.I.)	Coeff(95% C.I.)	Coeff(95%C.I.){Beta}*

**Variable**			

			

**% Of Mothers BMI > = 25**	5.51 (4.2, 6.8)		2.91 (1.7, 4.1){.401}

**% Less than High School**		1 (.37,.64)	.43 (.24, .51) {-.218}

**% Asian**		-.67 (-1.0,-.33)	-.42 (-.73,-.10){.403}

**% 50 years or older**		1.18 (.67,1.69)	.92 (.46, 1.4) {.275}

			

**Adjusted R^2^**	.52	.59	.75

**P value <**	.0001	0001	.0001

* Standardized regression coefficient

## Discussion

The results of this study suggest that data items pertaining to maternal height and weight included on the newly revised US Standard Certificate of Live Birth may be useful in public health research for the purposes of identifying neighborhoods with populations at relatively low or high risk for obesity. Philadelphia is somewhat unique in that local biannual health survey data were available to calculate estimates of the percentage of adults with BMI > = 25 for a significant number of the most highly populated census tracts. This provided the opportunity to explore the empirical relationship between BMI values derived from birth record data (pertaining to childbearing women) and those derived from survey data, representative of the entire adult population of the census tracts.

It seems reasonable to suggest that our findings may apply to other large urban areas where local survey or other data providing neighborhood-level obesity indicators may not be available. Further research is needed to confirm if this is in fact true.

The study reported here was exploratory in nature, intended as a proof-of-concept investigation of the potential value of public health data already being collected and widely available as a result of the nation's vital statistics record and US Census data systems. Given the positive results reported here, future research should explore if more advanced Geographic Information Systems (GIS) can be employed to improve on the definition of neighborhoods or communities (by combining census blocks or tracts, for example) in ways that may strengthen the evidence for the proxy value of data from the birth certificate records, or establish the generalizabilty of the findings here, which are admittedly limited to Philadelphia.

It is important to note that calculating census tract or other small area-level maternal BMI measures based on "observation" numbers that are relatively small (i.e., when the number of live births for any given area is small) may not be of much practical value because of statistical instability inherent in such calculations. In theory, where this problem of small numbers is involved, multiple years of birth certificate data could be combined to yield higher denominator values and thus increase the number of census tracts, neighborhoods, or other areas for which reliable measures could be calculated.

Birth certificate data are, of course, part of the US vital statistics system and are collected throughout the US, in collaboration with and under the auspices of state registrars and governments. The recently revised US Standard Certificate of Live Birth is being implemented gradually but has not yet been fully adopted by every state. Nor is it entirely clear which states tend to modify the Standard Certificate and in which ways. Since the maternal height and weight data elements are new to the revised Standard Certificate the findings here may be timely, and particularly relevant to city/county public health professional who have a growing need for obesity-related indicators at the small area or neighborhood levels

Finally, more broadly speaking, further research exploring potential "proxy value" of other data items appearing on birth certificate records would seem to be warranted. This is especially true as it pertains to priority health conditions or behaviors - such as smoking, hypertension, or diabetes -- with generally high impact on population health that are of considerable concern to local public health researchers, planners, advocates, and service providers. Public health researchers who have access to survey or other data sources could attempt to validate the use of birth certificate information to estimate the prevalence or risk of these morbidities at the small-area or neighborhood levels. To the extent they are able to do so, interest in using existing vital statistics data for the purposes of public health assessment and planning will expand and deepen, and thus the goal of identifying and validating sources of information that can be used to drive evidence-based policy and practice will be advanced.

## Competing interests

The authors declare that they have no competing interests.

## Authors' contributions

DW and JR conceived the design of the study, conducted the analyses and participated in all aspects in preparing the manuscript. JB and JC conducted literature reviews, assisted in preparation of the tables and writing the manuscript. All authors have read and approved the final manuscript.

## Author details

David A. Webb was with the Philadelphia Department of Public Health at the time of this writing and is now a Senior Research Associate in the Division of Adolescent Medicine at Children's Hospital of Philadelphia, Philadelphia, Pennsylvania, USA. Jessica M. Robbins is an epidemiologist at the Philadelphia Department of Public Health. Philadelphia, Pennsylvania, USA. Joan R. Bloch is an Assistant Professor in the College of Nursing and Health Professions and School of Public Health at Drexel University, Philadelphia, Pennsylvania, USA. Jennifer F. Culhane is a Senior Investigator in the Division of Adolescent Medicine at Children's Hospital of Philadelphia, Philadelphia, Pennsylvania, USA.
